# Transrenal DNA-based diagnosis of *Strongyloides stercoralis* (Grassi, 1879) infection: Bayesian latent class modeling of test accuracy

**DOI:** 10.1371/journal.pntd.0006550

**Published:** 2018-06-01

**Authors:** Alejandro J. Krolewiecki, Artemis Koukounari, Miryam Romano, Reynaldo N. Caro, Alan L. Scott, Pedro Fleitas, Ruben Cimino, Clive J. Shiff

**Affiliations:** 1 Instituto de Investigaciones de Enfermedades Tropicales, Universidad Nacional de Salta/CONIECT—Oran, Argentina; 2 Clinical Sciences Department, Liverpool School of Tropical Medicine, Liverpool, United Kingdom; 3 W. Harry Feinstone Department of Molecular Microbiology and Immunology, Bloomberg School of Public Health, Johns Hopkins University, Baltimore, MD, United States of America; 4 Catedra de Quίmica Biolόgica, Facultad de Ciencias Naturales, Universidad Nacional de Salta, Salta, Argentina; Christian Medical College, Vellore, INDIA

## Abstract

For epidemiological work with soil transmitted helminths the recommended diagnostic approaches are to examine fecal samples for microscopic evidence of the parasite. In addition to several logistical and processing issues, traditional diagnostic approaches have been shown to lack the sensitivity required to reliably identify patients harboring low-level infections such as those associated with effective mass drug intervention programs. In this context, there is a need to rethink the approaches used for helminth diagnostics. Serological methods are now in use, however these tests are indirect and depend on individual immune responses, exposure patterns and the nature of the antigen. However, it has been demonstrated that cell-free DNA from pathogens and cancers can be readily detected in patient’s urine which can be collected in the field, filtered *in situ* and processed later for analysis. In the work presented here, we employ three diagnostic procedures—stool examination, serology (NIE-ELISA) and PCR-based amplification of parasite transrenal DNA from urine–to determine their relative utility in the diagnosis of *S*. *stercoralis* infections from 359 field samples from an endemic area of Argentina. Bayesian Latent Class analysis was used to assess the relative performance of the three diagnostic procedures. The results underscore the low sensitivity of stool examination and support the idea that the use of serology combined with parasite transrenal DNA detection may be a useful strategy for sensitive and specific detection of low-level strongyloidiasis.

## Author summary

As international bodies focus efforts on control of the world’s neglected tropical diseases, the critical importance of accurate and sensitive diagnosis becomes a key factor. The problem arises when the infection load in a community is reduced to a level where the standard diagnostic methodologies are insufficiently sensitive to detect the residual infection in the community. There is a need to develop improved diagnostic strategies for many parasitic diseases. One of the more difficult to diagnose helminth parasites is the nematode *Strongyloides stercoralis*. We have introduced a new approach that detects parasite-specific cell free DNA in urine as a sensitive measure of parasite presence. In the work presented here, we compare the performance of parasitological, serological and urine/DNA-based diagnosis of *S*. *stercoralis* infection. Using a Bayesian Latent Class Analysis approach, we provide evidence for the enhanced utility of using both urine and blood for the diagnosis of this parasite.

## Introduction

The soil-transmitted parasitic nematode *Strongyloides stercoralis* is increasingly recognized as a significant human pathogen that deserves consideration for inclusion in the public health interventions that are underway to control other medically important soil transmitted helminths (STH) [1] such as *Ascaris lumbricoides*, *Trichuris trichiura* and the hookworms *Ancylostoma duodenale* and *Necator americanus* [2, 3]. The current STH control strategy does not include *S*. *stercoralis* as a target for chemotherapy. One of factors that has negatively influenced the inclusion of *S*. *stercoralis* as a target in the STH control efforts is the limited ability to diagnose an infection based on the standard, WHO-recommended, microscopic identification of larval parasites from stool samples [4]. While highly specific when carried out by experienced technical personnel, the sensitivity of this approach is compromised by the unpredictable, intermittent release of small numbers of larvae by adult parasites residing in the intestine [5]. Because this parasite is difficult to diagnose, the prevalence of *S*. *stercoralis* infection in many regions is largely unknown. There is a clear need for an improved approach for the diagnosis of *S*. *stercoralis* infection to define prevalence and the impact of intervention measures in the field.

In recognition of this need for better diagnostics, serological methods have been devised [6]. While significant advances have been made in terms of sensitivity, detection of specific antibodies is still subject to individual response as well as the antigens used in the tests to measure anti-*S*. *stercoralis* antibodies [7]. For increased specificity, nucleic acid-based diagnosis of *S*. *stercoralis* from stool samples using qPCR has been introduced. Although specific and amenable to multiplexing for the parallel detection of other pathogens [8, 9], this process has limitations, again, due to the intermittent presence of small numbers of *S*. *stercoralis* larvae passed in the feces of most patients. Additionally, collection of stool specimens in the field is labor intensive, costly and cumbersome.

The use of cell-free DNA in blood and other bodily fluids as biomarkers has gained wide acceptance in clinical laboratories. Cell-free DNA is being applied as a diagnostic marker for cancer, prenatal diagnosis and in infectious diseases, including parasitic diseases such as malaria, trypanosomiasis, leishmaniasis, schistosomiasis, strongylodiasis, and filariasis [10–12]. While most methods use blood, cell-free DNA is also readily detected in urine [12–14], saliva [15], stool [16], and sputum [17]. Cell-free DNA that is initially released into the blood can pass through the glomerular barrier and appear as transrenal DNAs in the urine [13] as small fragments of ~150–300 bp [18]. The advantages of transrenal DNA-based diagnosis of infectious disease include: (a) urine collection is non-invasive, (b) urine is easy and cheap to collect and process, and, in theory, (c) transrenal DNA does not depend on the stage of the parasite or the tissue site of infection.

In the current study, we employed a Bayesian Latent Class modeling approach to examine the diagnostic utility of three methodologically distinct diagnostic procedures—traditional comprehensive stool based parasitology, serology that employed a specific recombinant *S*. *stercoralis* larval antigen for the detection of anti-parasite antibodies, and a PCR-based analysis of urine for the detection of transrenal parasite DNA [19]. The Bayesian approach was used to address issues of misclassification of data because of different diagnostic targets and, importantly, the lack of a gold standard, to calibrate the diagnostic procedures. The selective modeling approach within a Bayesian framework also allowed us to establish a set of principled, evidence-based expectations about the diagnostic accuracy of the three methods and the overall prevalence, before incorporating the evidence from the observed data with the goal of improving the accuracy in estimates of regional prevalence of *S*. *stercoralis*.

## Methods

### Study design and participants

Our study was a cross-sectional assessment of diagnostic tests in rural and urban communities in Northwestern Argentina, in the Departments of Oran, San Martin and Rivadavia in Salta province. Eligible communities were those assigned to a sanitary intervention program carried out by the teams from Universidad Nacional de Salta, the Regional Sanitarian Development Association NGO, ADESAR, and the Provincial Ministries of Public Health and First Infancy. The objectives of this collaborative network were to provide medical care and epidemiological surveillance of intestinal parasitic infections in remote villages of the Chaco and Yunga geographic regions. A total of 359 participants provided a stool, a urine, and a serum sample.

### Ethics statement

The study was carried out and reported in accordance with the Standards for Reporting Diagnostic Accuracy (STARD-BLCM) guidelines [20]. Ethical approval for the study protocol and the informed consent form were obtained from Comité de Ética, Colegio Médico de Salta, Salta, Argentina dated 19 March 2015, and Johns Hopkins University (IRB number 6199) dated 30 April 2015. All participants provided written informed consent prior to sample collection. Parents or guardians provided informed consent on behalf of minor participants. All members of these communities were invited to participate and received anthelmintic treatment free of charge based on the results of stool analysis.

### Sample size assessment

Prior to the data collection, we performed a Monte Carlo simulation study that generated datasets of n = 400 observations 2000 times using a latent class analysis model in (Mplus 7 [21] [22]). The sample size was based on these simulations. In the model, based on an earlier study [15], we assumed that the true *Strongyloides* prevalence was 30%. Stool examination sensitivity was estimated to be 30%-40%. DNA and serological test sensitivity and DNA detection sensitivity was estimated to be 95% and 85%, respectively, based on previous work [23]. In the model we considered simulations involving four different tests. We assumed that the true prevalence of *Strongyloides* infection was 30% with stool examination sensitivity 70%, DNA and serological test 95% and antigen capture sensitivity 85% [24]. In this same model we also assumed stool examination specificity 100%, DNA specificity 98%, and serology specificity 75% and antigen capture specificity at 80% [24]. All parameters and standard error biases did not exceed 10% for any parameters in the model.

### Specimen collection and preparation

#### Stool

Each individual received a labeled sterile plastic container (no preservatives) and instructions on how to collect a stool sample. The stool samples were processed within 24 hours either in the laboratory at the Universidad Nacional de Salta in Salta city or at field laboratories assembled *ad-hoc* when in remote locations. The stool samples were analyzed using sedimentation-concentration, Harada Mori, McMaster´s and Baermann techniques for the microscopic identification of *S*. *stercoralis* and other helminths (Ascaris, hookworm, whipworm) [25]. Each participant provided a single stool sample.

#### Urine

Urine samples (~40 ml) were processed between one and two hours after collection by filtering through a conically-folded, 12.5 cm Whatman No. 3 filter disk. The filter disk was unfolded and dried at ambient temperature under a fly-proof cover, labeled for blind analysis, and stored in individual sealed plastic bags each with a desiccant sachet. The samples were sent to Johns Hopkins University in Baltimore for processing.

#### Serum

Blood samples (5 ml) were drawn by venipuncture, allowed to clot at ambient temperature and centrifuged. Serum was removed and aliquots stored at –20˚C. Anti-*S*. *stercoralis* antibody levels were analyzed with the in-house enzyme-linked immunosorbent assay (NIE-ELISA). NIE-ELISA detects IgG antibodies against a 31 kDa recombinant *S*. *stercoralis* L3 antigen, as has been described previously [7, 26, 27]. Patient sera were tested in duplicate and the mean of the results were used to calculated antibody concentration (in units/ml) by interpolation from a standard curve (eight serial dilutions of a known positive control serum). The cut off value for designating a sample as serum negative or serum positive (≥ 125 units/ml) was derived by construction of receiver operating curves (ROCs) calculated from ELISA results from 20 sera from stool positive patients and 20 sera from health normal patients from Salta Capital (non-endemic area for *S*. *stercoralis*).

#### DNA isolation and PCR amplification

The central regions of the filter papers containing the urine specimens were used to obtain 15 x 1.0 mm diameter discs using a paper punch. The discs were transferred to a 1.5 ml tube containing 800 μL of nuclease free water. After incubation at 95 ^0^ C for 10 minutes, the samples were subjected to gentle agitation at room temperature overnight. The tubes were then centrifuged at 8000 rpm for two minutes and the supernatant was removed and processed using QIAmpDNA Blood Mini Kit (Qiagen, MD) according to manufacturer’s protocol.

The forward primer (SSC-F) 5’ CTC AGC TCC AGT AAA GCA ACA G 3’ and reverse primer (SSC-R) 5’AGC TGA ATC TGG AGA GTG AAG A 3’ used to amplify a 124 base pair product from *S*. *stercoralis* dispersed repetitive sequence AY028262 [23, 28]. PCR amplification was done in a 15 μL volume with Taq 2X Mastermix (New England Biolabs, Ipswich, MA), 0.75 μL of 10 μM of each primer, 1–2 μL (2–4 ng/μL) DNA made to volume with PCR grade water (Sigma-Aldrich, St. Louis, Missouri). The amplification protocol was: Initial denaturation at 95°C for 10 minutes followed by 35 cycles of 95°C for 1 minute, 63°C for 90 seconds 72°C for 1 minute with final extension at 72°C for 10 minutes. In all instances specimens were run against a case positive control, and *S*. *stercoralis* genomic DNA to ensure effective amplification and to ensure identification of the diagnostic band. Negative controls were filtered urine from non-infected donors and no-DNA/nuclease-free water. The PCR product was resolved on a 2% agarose gel stained with Ethidium Bromide (Sigma-Aldrich, St. Louis, Missouri). The origin of the PCR product amplified from the urine samples was confirmed by sequencing. All specimens were run through duplicate reactions and any anomalies were repeated to confirm the status of the sample.

### Bayesian Latent Class Analysis (LCA)

In the absence of a gold standard for diagnosis of this infection and to take into account the inherent data misclassification, we fitted a latent class model using a Bayesian approach to assess the performance of the three diagnostic procedures used in this study. The basic idea behind the Bayesian approach is that all unknown quantities/model parameters such as the true *sensitivity* and *specificity* of each diagnostic test as well as the *prevalence* of the infection are believed to have a distribution that captures uncertainty about these parameter values. This uncertainty is captured by a distribution that is defined *before* observing the data and is called the *prior distribution* or *prior*. Prior data are derived from previous information from publications or experience in the field [7, 15]. Next, the observed evidence (i.e. the actual data) is expressed in terms of the *likelihood function* of the actual data. The actual data likelihood is then used to weigh the prior and this product yields the *posterior distribution*. Thus, the posterior distribution is a parameter comprised of the prior distribution and the likelihood function. Such a process allows simultaneous inferences to be made on all model parameters [29].

In our study, for each model parameter, the particular beta prior density was selected by matching the center of the range of the mean of the beta distribution according to Joseph et al. [29]. For stool sensitivity and specificity, we assumed a range of 20–40% (mean = 30%; beta parameters a = 1.9, b = 4.444) and a range of 95–100% (mean = 97.5%; beta parameters: a = 420.3, b = 10.7), respectively [7, 30]. For NIE-ELISA serology, we assumed a priori sensitivity of 81–88%, (mean = 84.5%; beta parameters a = 45.7, b = 15.1) and a specificity of 71–81%, (mean = 76%; beta parameters a = 68.2, b = 21.5) [26, 31]. For the diagnostic sensitivity and specificity for PCR DNA as well as prevalence by age groups we assumed non-informative priors which correspond to beta parameters a and b = 1. To account for age in the model for the prevalence of *Strongyloides* infection, <15 years old were considered children and ≥15 years represented adolescents and adults. As a sensitivity analysis, we also changed input values by 10% in each afore-mentioned prior, to evaluate the impact of priors on model outputs. As the examined tests in the present study are based on different biological measurements, we have assumed that they are not correlated to any substantial extent and thus that they are conditionally independent on the latent infection status (i.e. the latent class in the fitted model). The software we used to fit such a model was WinBUGS [32]. Multiple chains were run and results examined to ensure convergence.

### Additional statistics

The percent total agreement between PCR and NIE-ELISA serology results was calculated and Cohn’s kappa statistic was used to assess the overall agreement in results [33]. Analyses were done using the ‘irr’ package in R. (https://cran.r-project.org/web/packages/irr/irr.pdf)

## Results

### Comparison of test results

Table 1 outlines the distribution of the 359 participants in the study by age group (<15 years and ≥15 years), sex, and environmental context (rural vs urban). In the patients examined, 222/359 (62%) were positive for one or more of the diagnostic tests (Table 2). Serology and the transrenal DNA detection assays defined prevalence of 38% and 31%, respectively. In contrast, stool examination identified only ~8% of the participants as harboring an infection with *S*. *stercoralis*. There were no significant differences in infection status by any of the demographic categories used in this study.

**Table 1:** Patient demographics.

Rural				Urban			
<15 years		≥15 years		< 15 years		≥15 years	
Male	Female	Male	Female	Male	Female	Male	Female
53 (14.76)*	70 (19.50)	32 (8.91)	78 (21.73)	50 (13.93)	64 (17.83)	3 (0.84)	9 (2.51)

*percentage of total participants

**Table 2:** Prevalence of *Strongyloides stercoralis* infection obtained by Urine-based DNA detection and NIE-ELISA serology and coprology.

Diagnostic test	Number positive	Number negative	Total	% Prevalence (95% CI)
DNA	110	249	359	30.4 (25.6–35.4)
NIE-ELISA	134	225	359	36.7 (28.6–38.6)
Stool	30	329	359	8.4 (5.5–11.2)

**Fig 1:**
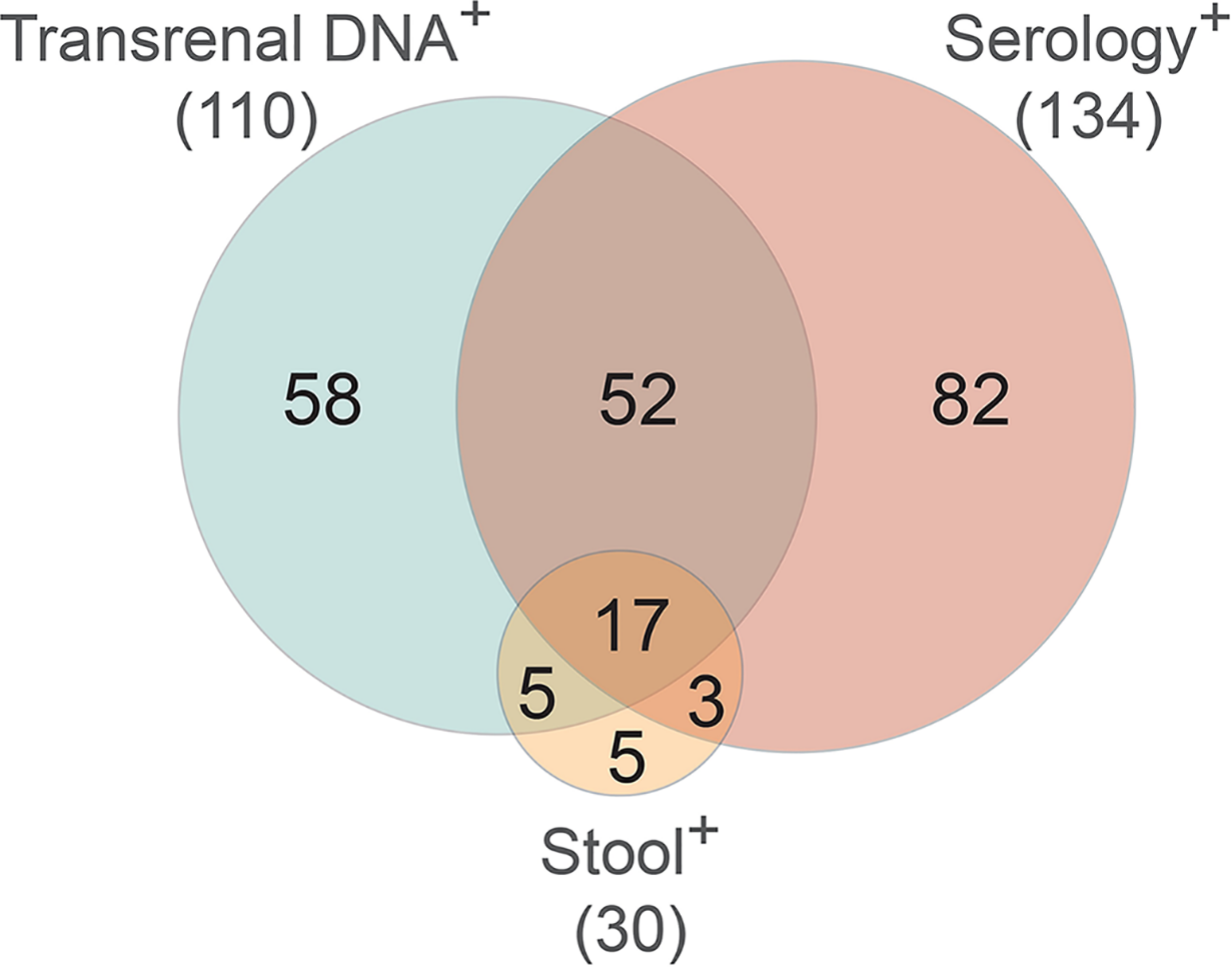
Venn diagram comparing the patient distribution of the 222 positive results of the transrenal DNA PCR, NEI ELISA serology and stool examination assays measuring *Strongyloides stercoralis* infection status.

The concordance in the assay outcomes between stool examination, serology, and the transrenal DNA test was evaluated (Fig 1). While ~53% of the participants were seropositive or transrenal DNA positive, only ~15% (52/359) were double positive for antibodies and transrenal DNA. Of the 359 samples examined total percent agreement between DNA and serology was only 61%. The kappa statistic was 0.131 with p = 0.0122 indicating the poor agreement between the two methods. Of the 30 patients who had detectable levels of parasites in their stool samples, 20 (66%) and 22 (73%) were positive by serological or transrenal DNA analysis, respectively. Over half of the stool-positive patients were also positive for serology and transrenal DNA (~4% of all patients). Therefore, nearly 70% of the seropositive participants tested negative for detectable amounts of parasite DNA in their urine and ~60% of the patients who were DNA positive had no detectable antibodies that bound to epitopes on the 31 kDa *S*. *stercoralis* L3 antigen.

### Bayesian Latent Class Analysis (LCA)

Table 3 contains the results from the Bayesian LCA model estimates (i.e. posterior medians and 95% Credible Intervals (CrI), which are the Bayesian analogs of confidence intervals) for sensitivity and specificity for each of the three diagnostic tests and the prevalence of *S*. *stercoralis* infection for the two age groups. The *S*. *stercoralis* infection prevalence in persons <15 years was estimated as 13.5% (95% CrI 5.9–24.8) and for age ≥15 years this was estimated to be 19.8% (95% CrI 10.7–34.2). These estimates are based on Bayesian latent class modeling of collective values between the three diagnostic tests having taken into account associated measurement error from each test, not on the results of any one single test, and thus they are more accurate than the empirically calculated prevalence in Table 2. The estimate of sensitivity for serology slightly exceeded the estimate of the diagnostic sensitivity for urine-based PCR, but their 95% corresponding credible intervals overlapped, suggesting that the diagnostic performances of these two tests were similar. Specificity of urine based-PCR was estimated to be slightly higher than that estimated for serology, but, again, the 95% credible intervals overlapped with the corresponding estimate for serology. There was no substantial change in these results when the priors were altered by 10%. The Bayesian modeling results confirm the low sensitivity (43.6: 95% CrI: 25.7 to 70.4) and high specificity (97.9; 95% CrI: 96.5 to 98.9) of stool examination for *S*. *stercoralis* infection.

**Table 3:** Bayesian LCA estimates of sensitivity and specificity and *Strongyloides stercoralis* prevalence with 95% Credible Intervals (CrIs) for results of three diagnostic tests (n = 359).

Diagnostic indicator	Bayesian LCA Sensitivity	Bayesian LCA Specificity
Serology	76.7 (67.1 to 85.1)	71.6 (65.7 to 77.4)
Stool	43.6 (25.7 to70.4)	97.9 (96.5 to 98.9)
PCR in urine	74.7 (53.8 to 91.8)	77.1 (71.7 to 83.7)
Bayesian LCA Model based *S*. *stercoralis* prevalence in younger age group (< = 15 years old)	13.5 (5.9 to 24.8)	
Bayesian LCA Model based *S*. *stercoralis* prevalence in older age group (>15 years old)	19.8 (10.7 to 34.2)	

## Discussion

Soil-transmitted and other helminth infections are of increasing global importance and are the focus of several wide spread mass drug administration efforts to reduce the level of morbidity inflicted on endemic populations by these parasites [2, 3, 34]. As these programs progress and the prevalence and intensity of infection declines because of these interventions, it is imperative to employ diagnostic strategies with increasing sensitivity and specificity to monitor and identify lingering infections. Decisions to prematurely suspend regional intervention efforts that are made based on the results of diagnostic tests that provide inaccurate assessments of prevalence and intensity are likely to undermine both short-term and long-term programmatic goals. Indeed, models indicate that helminth control programs that terminate prior to a solid control of transmission will result in reemergence and spread of the parasite into susceptible populations with detrimental public health consequences [35, 36]. Given the limited sensitivity of many of the standard methods used to monitor the prevalence of helminth infections, it is time to revise the diagnostic strategies for these parasites.

The goal of the work presented here was to determine if the detection of parasite-derived transrenal DNA has the potential to enhance the sensitivity of diagnosing *S*. *stercoralis* infection over an established and widely used serological assay or the standard parasitological stool analysis. Although it is clear from this work that detection of transrenal DNA and serology have an advantage over conventional stool analysis for the identification of infection, the relative merits of transrenal DNA and serological analysis are more difficult to conclude. While each test identified approximately the same number of participants as infected with *S*. *stercoralis*, only about 22% (53/243) were positive for both assays. It is tempting to conclude that direct detection of a *S*. *stercoralis*-derived molecule (transrenal DNA) is superior to the indirect measure of detecting antibodies that recognize a restricted set of epitopes associated with a single, stage-restricted parasite protein. However, in the absence of a ‘gold standard’ test, or set of reagents against which the accuracy of these two tests can be measured, such a determination cannot be made. The impact that a lack of gold standard tests has had on the development of molecular-based parasite diagnostics has been expertly reviewed elsewhere [29, 37–39]. The absence of a gold standard has prompted us [23] and others [29, 37] to employ Bayesian latent class modeling to generate estimates of specificity and sensitivity for parasite diagnostic tests. For the *S*. *stercoralis* diagnostic tests used in this study, latent class analysis confirms the low sensitivity of stool examination and concludes that the diagnostic performance of the NIE ELISA and transrenal DNA tests were similar in terms of diagnostic sensitivity and specificity (Table 3).

The limited concordance of the results from the serological and transrenal DNA tests can be of significant importance when MDA control efforts are evaluated. The low concordance may be due, in part, to the single molecule focus of these two assays. The NIE ELISA uses a recombinant form of a 31 kDa molecule expressed by infective *S*. *stercoralis* larvae [27] and was chosen for its favorable sensitivity and specificity profile [26] as well as its performance in clinical settings [7, 31]. The demonstrated utility of the NIE ELISA notwithstanding, both the sensitivity and specificity of this assay would likely benefit from the strategic inclusion of additional parasite molecules expressed by somatic cells of adults or released components of the parasite’s excretory/secretory products. Likewise, the transrenal DNA assay targets a single repeat sequence, the absence of which does not infer a negative diagnosis [23]. While it is possible that the clinical and/or parasitological status of certain patients preclude the passing parasite-derived transrenal DNAs, it is also likely that *Strongyloides* DNA was present in the urine but derived from other regions of the parasite’s genome. Identifying these additional transrenal sequences would provide an opportunity to devise a multiplex assay that amplifies several transrenal DNAs to enhance the diagnostic sensitivity and specificity of this approach.

The estimates for the half-life of cell-free DNA in the blood of humans range between 4 minutes and 12 hours (reviewed in [40]). Assuming that the proximate source of transrenal DNAs is the cell-free DNA in the blood, this short half-life indicates that detection of *Strongyloides*-derived DNA in the urine is measuring an ongoing infection. This rapid decay in the blood also suggests that testing for the presence of transrenal DNAs could be a sensitive tool to measure the efficacy of chemotherapeutic elimination of the parasite. In support of the utility of using transrenal DNAs as a marker of successful chemotherapy, Ibironke et al. [41] demonstrated that *Schistosoma haematobium* transrenal DNA was no longer detectable 14 days after treatment with praziquantel.

Diagnostic approaches that can accurately assess changes in disease burden and the impact of chemotherapeutic/public health for programs that are at different levels of control (breaking transmission, elimination, or post-elimination) are critical for strategic decision making. Following multiple rounds of treatment, MDA programs require highly sensitive assays to identify hot spots of residual transmission. In most cases, there is an unmet need to replace microscopy, which is not sufficiently sensitive to detect these low-level residual infections. At this time, no single nucleic acid, antigen detection or antibody approach appears to be able to provide an appropriately high-resolution picture of infection status. Given this, it may be time to consider coordinating the results of two or more molecular based assays for the diagnosis of STH’s, including strongyloidiasis. The results presented here suggest that the combined use of assays that detect transrenal DNA and antibodies may be a useful approach.

## References

[1] Krolewiecki AJ, Lammie P, Jacobson J, Gabrielli AF, Levecke B, Socias E, et al A public health response against Strongyloides stercoralis: time to look at soil-transmitted helminthiasis in full. PLoS Negl Trop Dis. 2013;7(5):e2165 doi: 10.1371/journal.pntd.0002165 ; PubMed Central PMCID: PMC3649958. 23675541 10.1371/journal.pntd.0002165 PMC3649958

[2] WHO. Soil-transmitted helminthiases: eliminating soil-transmitted helminthiases as a public health problem in children: progress report 2001–2010 and strategic plan 2011–2020. 2012. p. 89.

[3] WHO. Preventive chemotherapy to contrl soil-transmitted helminth infectons in at-risk populations groups. Geneva: 2017. 29578660

[4] WHO. Helminth control in school age children: a guide for managers of control programmes. 2nd ed. Geneva: WHO; 2011. p. 90.

[5] Requena-Mendez A, Chiodini P, Bisoffi Z, Buonfrate D, Gotuzzo E, Munoz J. The laboratory diagnosis and follow up of strongyloidiasis: a systematic review. PLoS Negl Trop Dis. 2013;7(1):e2002 Epub 2013/01/26. doi: 10.1371/journal.pntd.0002002 ; PubMed Central PMCID: PMCPMC3547839. 23350004 10.1371/journal.pntd.0002002 PMC3547839

[6] Bisoffi Z, Buonfrate D, Montresor A, Requena-Mendez A, Munoz J, Krolewiecki AJ, et al *Strongyloides stercorali*s: A Plea for Action. PLoS Negl Trop Dis. 2013;7(5):e2214 doi: 10.1371/journal.pntd.0002214 23675546 10.1371/journal.pntd.0002214 PMC3649953

[7] Bisoffi Z, Buonfrate D, Sequi M, Mejia R, Cimino RO, Krolewiecki AJ, et al Diagnostic accuracy of five serologic tests for Strongyloides stercoralis infection. PLoS Negl Trop Dis. 2014;8(1):e2640 doi: 10.1371/journal.pntd.0002640 ; PubMed Central PMCID: PMC3890421. 24427320 10.1371/journal.pntd.0002640 PMC3890421

[8] Pilotte N, Papaiakovou M, Grant JR, Bierwert LA, Llewellyn S, McCarthy JS, et al Improved PCR-Based Detection of Soil Transmitted Helminth Infections Using a Next-Generation Sequencing Approach to Assay Design. PLoS Negl Trop Dis. 2016;10(3):e0004578 doi: 10.1371/journal.pntd.0004578 ; PubMed Central PMCID: PMC4814118. 27027771 10.1371/journal.pntd.0004578 PMC4814118

[9] Cimino RO, Jeun R, Juarez M, Cajal PS, Vargas P, Echazu A, et al Identification of human intestinal parasites affecting an asymptomatic peri-urban Argentinian population using multi-parallel quantitative real-time polymerase chain reaction. Parasit Vectors. 2015;8:380 Epub 2015/07/18. doi: 10.1186/s13071-015-0994-z ; PubMed Central PMCID: PMCPMC4504406. 26183074 10.1186/s13071-015-0994-z PMC4504406

[10] Wagner J. Free DNA—new potential analyte in clinical laboratory diagnostics? Biochemia medica. 2012;22(1):24–38. ; PubMed Central PMCID: PMC4062320. 22384517 10.11613/bm.2012.004 PMC4062320

[11] Weerakoon KG, McManus DP. Cell-Free DNA as a Diagnostic Tool for Human Parasitic Infections. Trends Parasitol. 2016;32(5):378–91. doi: 10.1016/j.pt.2016.01.006 . 26847654 10.1016/j.pt.2016.01.006

[12] Yu J, Gu G, Ju S. Recent advances in clinical applications of circulating cell-free DNA integrity. Laboratory medicine. 2014;45(1):6–11. . 24719978 10.1309/lmkkox6ujzqgw0ea

[13] Botezatu I, Serdyuk O, Potapova G, Shelepov V, Alechina R, Molyaka Y, et al Genetic analysis of DNA excreted in urine: a new approach for detecting specific genomic DNA sequences from cells dying in an organism. Clinical chemistry. 2000;46(8 Pt 1):1078–84. . 10926886

[14] Ibironke OA, Phillips AE, Garba A, Lamine SM, Shiff C. Diagnosis of Schistosoma haematobium by detection of specific DNA fragments from filtered urine samples. Am J Trop Med Hyg. 2011;84(6):998–1001. Epub 2011/06/03. doi: 10.4269/ajtmh.2011.10-0691 ; PubMed Central PMCID: PMCPMC3110375. 21633040 10.4269/ajtmh.2011.10-0691 PMC3110375

[15] Jiang WW, Masayesva B, Zahurak M, Carvalho AL, Rosenbaum E, Mambo E, et al Increased mitochondrial DNA content in saliva associated with head and neck cancer. Clin Cancer Res. 2005;11(7):2486–91. doi: 10.1158/1078-0432.CCR-04-2147 . 15814624 10.1158/1078-0432.CCR-04-2147

[16] Diehl F, Schmidt K, Durkee KH, Moore KJ, Goodman SN, Shuber AP, et al Analysis of mutations in DNA isolated from plasma and stool of colorectal cancer patients. Gastroenterology. 2008;135(2):489–98. doi: 10.1053/j.gastro.2008.05.039 ; PubMed Central PMCID: PMC2820386. 18602395 10.1053/j.gastro.2008.05.039 PMC2820386

[17] van der Drift MA, Prinsen CF, Hol BE, Bolijn AS, Jeunink MA, Dekhuijzen PN, et al Can free DNA be detected in sputum of lung cancer patients? Lung cancer. 2008;61(3):385–90. doi: 10.1016/j.lungcan.2008.01.007 . 18313165 10.1016/j.lungcan.2008.01.007

[18] Su YH, Wang M, Brenner DE, Ng A, Melkonyan H, Umansky S, et al Human urine contains small, 150 to 250 nucleotide-sized, soluble DNA derived from the circulation and may be useful in the detection of colorectal cancer. The Journal of molecular diagnostics: JMD. 2004;6(2):101–7. doi: 10.1016/S1525-1578(10)60497-7 ; PubMed Central PMCID: PMC1867475. 15096565 10.1016/S1525-1578(10)60497-7 PMC1867475

[19] Lodh N, Naples JM, Bosompem KM, Quartey J, Shiff CJ. Detection of parasite-specific DNA in urine sediment obtained by filtration differentiates between single and mixed infections of Schistosoma mansoni and S. haematobium from endemic areas in Ghana. PLoS One. 2014;9(3):e91144 doi: 10.1371/journal.pone.0091144 ; PubMed Central PMCID: PMC3954594. 24632992 10.1371/journal.pone.0091144 PMC3954594

[20] Kostoulas P NS, Branscum AJ, Johnson WO, Dendukuri N, Dhand NK, Toft N, Gardner IA. STARD-BLCM: Standards for the Reporting of Diagnostic accuracy studies that use Bayesian Latent Class Models. Prev Vet Med. 2017;138:37–47. doi: 10.1016/j.prevetmed.2017.01.006 28237234 10.1016/j.prevetmed.2017.01.006

[21] Muthen LKaM B.O. How to Use a Monte Carlo Study to Decide on Sample Size and Determine Power,. Structural Equation Modelling: A Multidisiplinary Journal. 2002;9(4):599 doi: 10.1207/S15328007SEM0904_8

[22] Muthen LK, Muthen B. Mplus User's Guide. 6th ed Los Angeles, CA: Muthen and Muthen; 2010 2010.

[23] Lodh N, Caro R, Sofer S, Scott A, Krolewiecki A, Shiff C. Diagnosis of Strongyloides stercoralis: Detection of parasite-derived DNA in urine. Acta tropica. 2016;163:9–13. doi: 10.1016/j.actatropica.2016.07.014 ; PubMed Central PMCID: PMC5117362. 27456935 10.1016/j.actatropica.2016.07.014 PMC5117362

[24] Sykes AM, McCarthy JS. A coproantigen diagnostic test for Strongyloides infection. PLoS Negl Trop Dis. 2011;5(2):e955 doi: 10.1371/journal.pntd.0000955 21347447 10.1371/journal.pntd.0000955 PMC3035667

[25] Garcia L. Diagnostic Medical Parasitology. 4 ed Washington. DC: ASM Press; 2001 2001.

[26] Krolewiecki AJ, Ramanathan R, Fink V, McAuliffe I, Cajal SP, Won K, et al Improved diagnosis of Strongyloides stercoralis using recombinant antigen-based serologies in a community-wide study in northern Argentina. Clinical and vaccine immunology: CVI. 2010;17(10):1624–30. doi: 10.1128/CVI.00259-10 ; PubMed Central PMCID: PMC2952987. 20739501 10.1128/CVI.00259-10 PMC2952987

[27] Ravi V, Ramachandran S, Thompson RW, Andersen JF, Neva FA. Characterization of a recombinant immunodiagnostic antigen (NIE) from Strongyloides stercoralis L3-stage larvae. Mol Biochem Parasitol. 2002;125(1–2):73–81. . 12467975 10.1016/s0166-6851(02)00214-1

[28] Lodh N, Caro N, Sofer S, Scott A, Krolewiecki A, Shiff C. Diagnosis of *Strongyloides stercoralis*: Detection of parasite-derived DNA in urine. Acta Tropica. 2016;163:9–13. doi: 10.1016/j.actatropica.2016.07.014 27456935 10.1016/j.actatropica.2016.07.014 PMC5117362

[29] Joseph L, Gyorkos TW, Coupal L. Bayesian estimation of disease prevalence and the parameters of diagnostic tests in the absence of a gold standard. Am J Epidemiol. 1995;141(3):263–72. Epub 1995/02/01. . 7840100 10.1093/oxfordjournals.aje.a117428

[30] Bisoffi Z, Buonfrate D, Angheben A, Boscolo M, Anselmi M, Marocco S, et al Randomized clinical trial on ivermectin versus thiabendazole for the treatment of strongyloidiasis. PLoS Negl Trop Dis. 2011;5(7):e1254 doi: 10.1371/journal.pntd.0001254 ; PubMed Central PMCID: PMC3144183. 21814588 10.1371/journal.pntd.0001254 PMC3144183

[31] Buonfrate D, Sequi M, Mejia R, Cimino RO, Krolewiecki AJ, Albonico M, et al Accuracy of five serologic tests for the follow up of Strongyloides stercoralis infection. PLoS Negl Trop Dis. 2015;9(2):e0003491 Epub 2015/02/11. doi: 10.1371/journal.pntd.0003491 ; PubMed Central PMCID: PMCPMC4323101. 25668740 10.1371/journal.pntd.0003491 PMC4323101

[32] Lunn D, Thomas A., Best N. Spiegelhalter D. WinBUGS—A Bayesian Modelling Framework: concepts, structure and extensibility. Statistics nas Computing. 2000;10::325–37.

[33] Cohen J. A coefficient of agreement fro nomincal scales. Educational and Psychological Measurement. 1960;20(1):37–46. doi: 10.1177/001316446002000104

[34] Molyneux DH. The London Declaration on Neglected Tropical Diseases: 5 years on. Trans R Soc Trop Med Hyg. 2017 doi: 10.1093/trstmh/trw082 . 28115685 10.1093/trstmh/trw082

[35] Anderson R, Farrell S, Turner H, Walson J, Donnelly CA, Truscott J. Assessing the interruption of the transmission of human helminths with mass drug administration alone: optimizing the design of cluster randomized trials. Parasit Vectors. 2017;10(1):93 doi: 10.1186/s13071-017-1979-x ; PubMed Central PMCID: PMC5316156. 28212667 10.1186/s13071-017-1979-x PMC5316156

[36] Mitchell KM, Mutapi F, Mduluza T, Midzi N, Savill NJ, Woolhouse ME. Predicted impact of mass drug administration on the development of protective immunity against Schistosoma haematobium. PLoS Negl Trop Dis. 2014;8(7):e3059 doi: 10.1371/journal.pntd.0003059 ; PubMed Central PMCID: PMC4117464. 25079601 10.1371/journal.pntd.0003059 PMC4117464

[37] Nikolay B, Brooker SJ, Pullan RL. Sensitivity of diagnostic tests for human soil-transmitted helminth infections: a meta-analysis in the absence of a true gold standard. Int J Parasitol. 2014;44(11):765–74. doi: 10.1016/j.ijpara.2014.05.009 ; PubMed Central PMCID: PMC4186778. 24992655 10.1016/j.ijpara.2014.05.009 PMC4186778

[38] O'Connell EM, Nutman TB. Molecular Diagnostics for Soil-Transmitted Helminths. Am J Trop Med Hyg. 2016;95(3):508–13. doi: 10.4269/ajtmh.16-0266 ; PubMed Central PMCID: PMC5014250. 27481053 10.4269/ajtmh.16-0266 PMC5014250

[39] Tarafder MR, Carabin H, Joseph L, Balolong E Jr., Olveda R, McGarvey ST. Estimating the sensitivity and specificity of Kato-Katz stool examination technique for detection of hookworms, Ascaris lumbricoides and Trichuris trichiura infections in humans in the absence of a 'gold standard'. Int J Parasitol. 2010;40(4):399–404. doi: 10.1016/j.ijpara.2009.09.003 ; PubMed Central PMCID: PMC2829363. 19772859 10.1016/j.ijpara.2009.09.003 PMC2829363

[40] Khier S, Lohan L. Kinetics of circulating cell-free DNA for biomedical applications: critical appraisal of the literature. Future science OA. 2018;4(4):FSO295 doi: 10.4155/fsoa-2017-0140 ; PubMed Central PMCID: PMC5905581. 29682327 10.4155/fsoa-2017-0140 PMC5905581

[41] Ibironke O, Koukounari A, Asaolu S, Moustaki I, Shiff C. Validation of a new test for Schistosoma haematobium based on detection of Dra1 DNA fragments in urine: evaluation through latent class analysis. PLoS Negl Trop Dis. 2012;6(1):e1464 doi: 10.1371/journal.pntd.0001464 ; PubMed Central PMCID: PMC3250497. 22235360 10.1371/journal.pntd.0001464 PMC3250497

